# Halting NDM-producing enterobacteriaceae spread with the reactive infection control strategy: a real-world experience analyzed using a novel spatiotemporal epidemiologic risk measure (Epi-score) and whole-genome sequencing (WGS)

**DOI:** 10.1186/2047-2994-4-S1-O41

**Published:** 2015-06-16

**Authors:** K Marimuthu, O Ng, W Khong, E Xia, W Xu, Y Teo, E Tan, RT Ong, D Lye, A Chow, P Krishnan, B Ang

**Affiliations:** 1 tan Tock Seng Hospital, Singaore; 2 National University of Singapore, Singapore

## Introduction

Guidelines recommend screening of epidemiologically linked patients to contain the spread of NDM-producing enterobacteriaceae (NDM-EB).

## Objectives

We assessed the effectiveness of this strategy in controlling the spread of NDM-EB using Epi-score and whole-genome sequencing (WGS).

## Methods

This study was conducted at Tan Tock Seng Hospital (TTSH) between September 2010 and December 2011. TTSH implemented a reactive infection control strategy which constituted pre-emptive cohorting and rectal surveillance of patients with epidemiological linkage (contacts) to patients with NDM-EB isolated from clinical cultures (index). A clinical transmission model was produced for NDM-EB patients both from clinical and surveillance cultures based on epidemiological relatedness. The Epi-score graded epidemiologic-relatedness from 0 (unrelated) to 4 (very related), based on spatiotemporal ward overlap (2 points), shared medical teams (1 point), and shared medical department (1 point). This was the compared with a molecular transmission model which was produced using WGS of all NDM-EB isolates with core-genome single-nucleotide polymorphism analysis after excluding recombinant sites.

## Results

A total of six index clinical NDM-EB were detected (patients 1-3,5,7-8) (5 urine and 1 bile). Contact screening as part of reactive strategy involved 436 patients, of which 2 (patients 4, 6) were newly-detected NDM-EB carriers. Epi-score distributions: 3 points (patients 1-4,2-3,5-6); 2 points (patients 1-5,4-5), 1 point (patients 1-7,5-8,6-8). Of the 3 point pairs, one (5-6) was confirmed direct transmission by whole-genome phylogenetic analysis (4 SNVs). None of the other patients were identified by WGS as direct transmission. In six (75%) isolates, NDM was carried on plasmid pTR3 which is unique to Singapore.

## Conclusion

Only one direct ward transmission pair (confirmed by whole-genome-sequencing) was detected by the reactive strategy. The Epi-score performed well in classifying the direct ward transmission pair in the highest relatedness category. Most (75%) NDM-producing EB originated in Singapore, with possibly 25% of NDM-producing EB from overseas.

## Disclosure of interest

None declared.

